# Dose escalation of tolinapant (ASTX660) in combination with standard radical chemoradiotherapy in cervical cancer : a study protocol for a phase 1b TiTE-CRM clinical trial (CRAIN) in UK secondary care centres

**DOI:** 10.1186/s12885-024-12310-w

**Published:** 2024-06-07

**Authors:** Peter Hoskin, Marina Lee, Denise Dunkley, Mary Danh, Robin Wickens, Geoff Saunders, Josh Northey, Simon Crabb, Vicky McFarlane, Azmat Sadozye, Rachel Cooper, Tony Mathew, Kate Haslett, Kim Reeves, Rachel Reed, Kamilla Bigos, Kaye J. Williams, Emily Rowling, Ananya Choudhury, Sonia Dancer, Deb Smith, Gareth Griffiths

**Affiliations:** 1 Division of Cancer Sciences, University of Manchester, Manchester, UK; 2 Southampton Clinical Trials Unit, University of Southampton, Southampton, UK; 3 University Hospital Southampton NHS Foundation Trust, Southampton, UK; 4 The Beatson West of Scotland Cancer Centre, Glasgow, UK; 5 The Leeds Teaching Hospitals NHS Trust, Leeds, UK; 6 Sheffield Teaching Hospitals NHS Foundation Trust, Sheffield, UK; 7 The Christie NHS Foundation Trust, Manchester, UK; 8 Division of Pharmacy and Optometry, University of Manchester, Manchester, UK

**Keywords:** Cervical cancer, Tolinapant, Chemoradiotherapy, Cisplatin, Phase I

## Abstract

**Background:**

Cervical cancer is the fourth most common cancer in women, with an estimated 342,000 deaths worldwide in 2020. Current standard of care in the UK for locally advanced cervical cancer is concurrent chemoradiotherapy with weekly cisplatin, yet 5-year overall survival rates are only 65% with a distant relapse rate of 50%. Inhibitors of Apoptosis Proteins (IAPs) are often overexpressed in cancer cells and associated with tumour progression and resistance to treatment. Tolinapant, developed by Astex Pharmaceuticals, is an IAP antagonist with an additional mechanism of action via down-regulation of NF-kB, an important regulator in cervical cancer. Preclinical studies performed using tolinapant in combination with cisplatin and radiotherapy showed inhibition of tumour growth and enhanced survival. There is therefore a strong rationale to combine tolinapant with chemoradiotherapy (CRT).

**Methods:**

CRAIN is a phase Ib open-label, dose escalation study to characterise the safety, tolerability and initial evidence for clinical activity of tolinapant when administered in combination with cisplatin based CRT. Up to 42 patients with newly diagnosed cervix cancer will be recruited from six UK secondary care sites. The number of participants and the duration of the trial will depend on toxicities observed and dose escalation decisions, utilising a TiTE-CRM statistical design. Treatment will constist of standard of care CRT with 45 Gy external beam radiotherapy given in 25 daily fractions over 5 weeks with weekly cisplatin 40mg/m^2^. This is followed by brachytherapy for which common schedules will be 28 Gy in 4 fractions high-dose-rate or 34 Gy in 2 fractions pulsed-dose-rate. Tolinapant will be administered in fixed dose capsules taken orally daily for seven consecutive days as an outpatient on alternate weeks (weeks 1, 3, 5) during chemoradiation. Dose levels for tolinapant which will be assessed are: 60 mg; 90 mg (starting level); 120 mg; 150 mg; 180 mg. Escalation will be guided by emerging safety data and decisions by the Safety Review Committee.

**Discussion:**

If this trial determines a recommended phase II dose and shows tolinapant to be safe and effective in combination with CRT, it would warrant future phase trials. Ultimately, we hope to provide a synergistic treatment option for these patients to improve outcome.

**Trial registrations:**

EudraCT Number: 2021-006555-34 (issued 30th November 2021); ISRCTN18574865 (registered 30th August 2022).

**Supplementary Information:**

The online version contains supplementary material available at 10.1186/s12885-024-12310-w.

## Background

Cervical cancer is the fourth most common cancer in women, with an estimated 342,000 deaths worldwide in 2020 [1]. Current standard of care in the UK for locally advanced cervical cancer is concurrent chemoradiotherapy (CRT) with weekly cisplatin, yet 5-year overall survival rates are only around 65% with a distant relapse rate of 50% [2]. This treatment is associated with long term side effects in around half of patients with up to 15% suffering from grade 3–4 toxicity [3].

Evasion of apoptosis is one of the hallmarks of cancer [4] and apoptosis is a key mechanism for programmed cell death that is dysregulated in many tumour types. Inhibitors of Apoptosis Proteins (IAPs) are key regulators of antiapoptotic and pro-survival signalling pathways, which are often overexpressed in cancer cells and associated with tumour progression and resistance to treatment [5]. Tolinapant is an IAP antagonist, which is chemically distinct from first generation peptidomimetic SMAC (second mitochondria-derived activator of caspase) mimetics and shows balanced inhibition across the subtypes of IAP: cIAP1, cIAP2 and XIAP [6]. A putative additional mechanism of action of tolinapant is via down-regulation of NF-kB which is an important regulator in cervical cancer.

Tolinapant, developed by Astex Pharmaceuticals, is a novel antagonist of cIAP1/2 and XIAP currently in clinical trials for treating advanced solid tumours i.e. head and neck squamous cell carcinoma (HNSCC), cervical carcinoma and other tumour types that are characterized by a molecular feature that may confer sensitivity to tolinapant, and various lymphomas. Studies using a panel of cervical carcinoma cell lines illustrated that tolinapant causes cIAP1 depletion and induces apopotosis that was enhanced when combined with radiation and cisplatin. Apoptosis correlated with reduced overall cell survival. Tolinapant-mediated enhancement of CRT was observed in in vivo using xenograft models of cervical carcinoma, with no excaberation of toxicity (manuscript in preparation, Astex Pharmaceuticals, data on file).

Further preclinical studies performed using tolinapant in combination with cisplatin and radiotherapy showed an inhibition of tumour growth and enhanced survival in multiple HPV+/- head and neck squamous cell carcinoma models. Additional data using a syngeneic mouse oral cancer model also showed tolinapant in combination induced tumour growth inhibition compared to RT alone and tolinapant alone [7].

There is therefore a strong rationale to combine tolinapant with CRT. Improvement in loco-regional control is closely related to survival improvement in cervical cancer hence this study has the potential to improve survival in a population of relatively young active women.

CRAIN is a phase 1b trial to establish the maximum tolerated dose and to determine the recommended phase II dose (RP2D) of tolinapant combined with cisplatin & radiotherapy (CRT) and to then determine whether there is a signal of efficacy sufficient to justify further study of the combination, in the primary treatment of cervical cancer, in later phase trials.

## Methods/design

CRAIN is a phase Ib open-label, dose escalation trial of tolinapant in combination with cisplatin based CRT in women with cancer of the cervix.

### Objectives

The CRAIN trial primary objective is to establish the maximum tolerated dose of tolinapant in combination with CRT and to determine a RP2D for a future phase II trial. The primary end point is dose limiting toxicities (DLTs).

Secondary objectives are to determine the safety and tolerability (using Common Terminology Criteria for Adverse Events [CTCAE] version 5), response rate (using Response Evaluation Criteria in Solid Tumors [RECIST] v1.1) and impact on planned delivery of CRT of the combination of CRT + tolinapant. Tertiary objectives included an evaluation of on-target effects of tolinapant and to explore tissue and liquid biomarkers which may predict response to tolinapant.

Translational objectives include the pharmacokinetics of tolinapant, identification of markers of therapy response, identification of predictors of response to CRT + tolinapant, identification of patients with hypoxic tumours and identification of genetic factors affecting treatment response.

### Study design

**Fig. 1:**
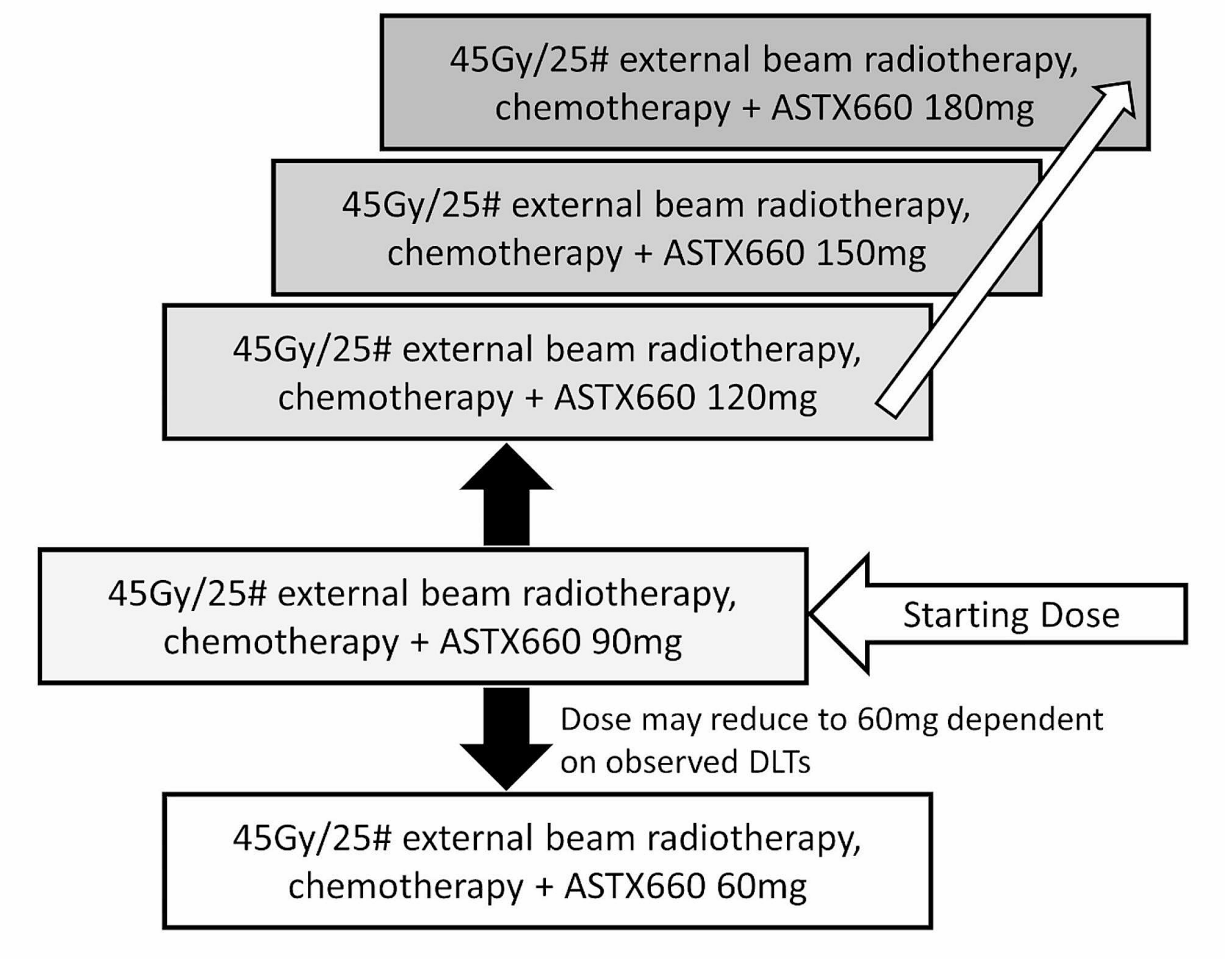
CRAIN trial schema

CRAIN is a phase 1b open-label, UK multi-centre study to characterise the safety and tolerability and initial evidence for clinical activity of tolinapant when administered in combination with cisplatin based CRT to women aged 16 or over with histologically proven adenocarcinoma or squamous cell carcinoma of the cervix. The trial uses a two-stage, time-to event continual reassessment method (TiTE-CRM) design to find the maximum tolerated safe dose (MTD) of tolinapant. The CRAIN trial schema (Fig. 1) details the study design.

#### Dose limiting toxicology

The DLT assessment period is 12 weeks from the start of treatment.

Any of the following events occurring after the first dose of tolinapant will constitute a DLT if, in the opinion of the investigator, the event is defined as definitely or probably related to tolinapant:

- Grade 4 neutropenia ≥ 7 days duration.
- Grade 3 or 4 febrile neutropenia (neutrophils < 1000/mm3 with a single temperature of > 38.3 °C or a sustained temperature of ≥ 38 °C for more than one hour AND/OR life-threatening consequences with urgent intervention indicated).
- Grade 3 or 4 neutropenia associated with a separate event of bacteriologically proven sepsis happening at the same time.
- Grade 3 or 4 thrombocytopenia.
- Death.

Any other grade 3 or 4 adverse event will constitute a DLT if, in the opinion of the investigator, the event is defined as definitely or probably related to tolinapant.

In all cases of a suspected DLT, clinical judgement will be the final arbiter as to whether the event should be categorised as such.

#### Setting

CRAIN will be run across six secondary care hospitals in the UK over 24 months.

#### Sample size and recruitment

The actual number of patients required and the duration of the trial will depend on any toxicities observed and dose escalation decisions. As a result of the stopping rules (see additional file 1), all simulated toxicity scenarios, using the stats package R, averaged 24–29 participants, while a maximum of 42 patients was deemed suffient to estimate the MTD and be able to recruit additional patients to ensure 18 are treated at the recommended phase II dose. In order to ensure adequate recruitment, the trial will include patients with a wide range of cervical cancer stages; including those with radiologically active pelvic nodes. In addition the chosen trial sites for CRAIN are geographically widespread throughout the UK and press releases are timed alongside key site openings to enhance recruitment.

### Ethical and regulatory aspects

The study received ethical approval from North West - Haydock Research Ethics Committee on 14th August 2022 (ref: 22/NW/0235) and has Health Research Authority (IRAS 1,004,372) and UK Medicines and Health Care Product Regulatory Agency (MHRA) approvals. Southampton Clinical Trials Unit (SCTU), a Cancer Research UK core funded and UK Clinical Research Collaboration registered Clinical Trials Unit (CTU), is coordinating the trial, sponsored by University Hospital Southampton NHS Foundation Trust.

### Study participants

CRAIN is currently recruiting women aged 16 or over with histologically proven adenocarcinoma or squamous cell carcinoma of the cervix stage IB2/IB3/IIA1/IIA2/IIB/IIIA/IIIB/IIIC1 suitable for radical treatment with radiotherapy and cisplatin, with a ECOG Performance Status 0–1. The full eligibility criteria are listed in Table 1.

**Table 1:** CRAIN trial eligibility criteria

Inclusion criteria	
1.	Histologically confirmed adenocarcinoma or squamous cell carcinoma of the cervix stage IB2/IB3/IIA1/IIA2/IIB/IIIA/IIIB/IIIC1* (using the revised 2018 FIGO staging classification for cervical cancer) *any stage IIIC1 patients must have treatment planned with the same volume and tissue constraints as node negative patients. In stage IIIA patients inguinal nodes may be included but patients with extended field volumes to include para-aortic nodes would not be eligible.
2.	Suitable for radical treatment with radiotherapy and cisplatin (using a standard dose of 45 Gy in 25 daily fractions over 5 weeks with weekly cisplatin 40 mg/m2 ).
3.	Adequate haematological parameters: Haemoglobin ≥ 90 g/L, Neutrophil count ≥ 1.5 × 10^9^/L, Platelets ≥ 100 × 10^9^/L
4.	Adequate biochemical parameters: Bilirubin ≤ 1.5 x ULN, AST and ALT ≤ 2.0 x ULN, ALP ≤ 2.5 x ULN, Lipase and Amylase ≤ 1.2 x ULN, Estimated GFR (calculated using the CKD-EPI formula or other accepted formula) or measured directly as ≥ 50 mL/min
5.	Age 16 years or over
6.	ECOG Performance Status of 0–1
7.	Willing and able to give written informed consent
**Exclusion criteria**	
1.	Previous pelvic radiotherapy
2.	Liver cirrhosis, or chronic liver disease Child-Pugh Class B or C
3.	Women who are pregnant or breast feeding (WOCBP must have a negative serum pregnancy test at screening)
4.	Patients of child-bearing potential who are not able to use a method of contraception as detailed in Sect. 4.6
5.	Any investigational medicinal product within 30 days prior to consent
6.	Major surgery within 30 days prior to enrolment
7.	Hypersensitivity to tolinapant, excipients of the drug product, or other components of the study treatment regimen
8.	Patients with known HIV infection
9.	Patients with known active hepatitis B virus (HBV; chronic or acute; defined as having a positive hepatitis B surface antigen [HBsAg] test) or hepatitis C. Patients with past HBV infection or resolved HBV infection (defined as the presence of hepatitis B core antibody and the absence of HBsAg) are eligible. Patients positive for hepatitis C virus (HCV) antibody are eligible only if polymerase chain reaction is negative for HCV RNA
10.	Coronary artery bypass graft, angioplasty, vascular stent, myocardial infarction, unstable arrhythmias, unstable angina, left bundle branch block, third degree heart block, pacemakers or congestive cardiac failure (New York Heart Association ≥ grade 2) within 6 months prior to enrolment
11.	Any patient who has received a live vaccine within 4 weeks of initiation of their treatment (COVID-19 vaccination is allowed).
12.	Conditions requiring systemic treatment with either corticosteroids (≥ 20 mg daily prednisolone or equivalent) or other immunosuppressive medications within 14 days of study drug administration
13.	Prior anticancer treatments or therapies within the indicated time window prior to first dose of study treatment (tolinapant), as follows: (a) Cytotoxic chemotherapy or radiotherapy within 3 weeks prior and any encountered treatment-related toxicities (excepting alopecia) not resolved to Grade 1 or less, (b) Skin directed treatments, including topicals and radiation within 2 weeks prior, (c) Monoclonal antibodies within 4 weeks prior and any encountered treatment-related toxicities not resolved to Grade 1 or less, (d) Small molecules or biologics (investigational or approved) within the longer of 2 weeks or 5 half-lives prior to study treatment and any encountered treatment-related toxicities not resolved to Grade 1 or less, (e) At least 6 weeks must have elapsed since CAR-T infusion and subjects must have experienced disease progression, and not have residual circulating CAR-T cells in peripheral blood (based on local assessment). Any encountered treatment-related toxicities must have resolved to Grade ≤ 1.
14.	Patients taking a QT prolonging agent (with the exception of palonesetron/Akynzeo when used as part of the chemotherapy antiemetic regime), see appendix for example list.
15.	Use of a concomitant medication which is a strong CYP3A4 inhibitor.
16.	Abnormal left ventricular ejection fraction (LVEF) of < 50% on echocardiogram (ECHO).
17.	History of long QTc syndrome or ventricular arrhythmias including ventricular bigeminy.
18.	Screening 12-lead electrocardiogram (ECG) with measurable QTc interval of ≥ 470 msec (according to either Fridericia’s or Bazett’s correction).
19.	Any other active malignancy.
20.	Any known active covid-19 infection at the time of consent.

### Study procedure

#### Informed consent

Consent to enter the trial must be sought from each participant only after a full explanation has been given, a Patient Information Sheet offered and time allowed for consideration. Signed participant consent must be obtained prior to any study specific procedures. Consent may only be obtained by staff authorized to do so on the delegation log.

##### Withdrawal criteria

Participants are free to withdraw consent from the study at any time without having to provide a reason. Where possible, participants who have withdrawn from trial treatment should remain in follow-up as per the trial schedule. Data and samples collected prior to participant withdrawal will still be used for trial analysis.

#### Screening

Following consent, patients undergo screening assessments that should be completed within 2 weeks of treatment, with the exclusion of the diagnostic/baseline scans, which can be done within 8 weeks prior to treatment. Assessments include physical examination, ECOG performance status, ECG, echocardiogram, serum biochemistry (including renal, liver, bone and thyroid profiles, plus random cortisol and glucose levels), haematology and serology (HBV and HCV). Concomitant medication and medical history will be recorded. In addition, women of childbearing potential (WOCBP) will undertake a pregnancy test.

#### Treatment and follow-up visits

Treatment will consist of standard of care CRT with 45 Gy external beam radiotherapy administered in 25 daily fractions over 5 weeks with once weekly cisplatin 40 mg/m2. RT visit assessments include ECOG performance status, routine bloods, pregnancy tests for WOCBP (weeks 3 & 5) and adverse event assessment (weeks 1, 3 & 5). Six PK samples will be taken across day 4 and day 5 of week 1.

Tolinapant will be administered in fixed dose capsules of 30 mg or 90 mg taken orally daily for seven consecutive days as an outpatient (followed by seven consecutive days off) prior to radiotherapy on alternate weeks (weeks 1, 3, 5) during chemoradiation. As tolinapant will be administered as an outpatient, and in order to assess IMP administration adherence, patient diaries will be reviewed and capsule returns monitored. Tolinapant can be taken with or without food, with the exception of PK days. On days with PK sampling, patients should refrain from eating (including soup) or drinking milk or juice for at least 2 h prior to ingesting study drug(s) and 2 h after ingesting study drug (4 h fasting window).

This is followed by standard of care brachytherapy. Assessments during brachytherapy visits will include physical exams, ECOG performance status, routine bloods (week 6 only) and adverse event assment (week 7 only).

Patients will then attend a follow-up visit at weeks 12 and 18, involving physical exams (including vaginal adhesions at week 18), ECOG performance status, adverse event assessment, MRI (week 18 only) and Disease Assessment Questions (week 18 only).

The Schedule of Events (Table 2) details the trial treatment schedule.

**Table 2:** Schedule of observations and procedures

	Screening Phase	Treatment Phase							Follow up Phase	
		External Beam RT Treatment Week 1	External Beam RT Treatment Week 2	External Beam RT Treatment Week 3	External Beam RT Treatment Week 4	External Beam RT Treatment Week 5	Brachytherapy Week 1	Brachytherapy Week 2	Follow up visit 1	Follow up visit 2
Trial Week^1^	-2-0 weeks	1	2	3	4	5	6	7	12 (± 1 week)	18 (± 1 week)
Informed consent^2^	x									
Inclusion / exclusion criteria	x									
Medical history	x									
Physical exam	x						x	x	x	X^3^
ECOG performance status	x	x	x	x	x	x	x	x	x	x
Electrocardiogram	x									
Echocardigram	x									
Serum biochemistry^4^	x	x	x	x	x	x	x			
Haematology^5^	x	x	x	x	x	x	x			
Serology – HBV and HCV	x									
Pregnancy test for WOCBP^6^	x			x		x				
Translational tissue samples^7^	x						x			
Translational blood samples	x	x	x	x	x	x	x	x	x	
Translational urine samples	x	x		x		x	x		x	x
Diagnostic imaging assessment (MRI)	X^8^					x				X
CT scan to assess for metastases^9^	x									
Disease Assessment Questions^10^	x					x				x
Adverse event assessment	x	x		x		x		x	x	x
Concomitant medication record	x								x	x
Tolinapant^11^		x		x		x				
PK sampling^12^		xxxxxx								
External beam radiotherapy (standard 5 week course)^13^		x	x	x	x	x				
Cisplatin (40 mg/m^2^)		x	x	x	x	x				
Brachytherapy^14^							x	x		

^1^Treatment weeks of CRT and tolinapant should always be scheduled to start on a Monday, cisplatin can be given on any day throughout the week

^2^Consent may be taken up to 14 days prior to Treatment day 1, week 1 and must be completed before any screening procedures take place

^3^Vaginal adhesions should be assessed as part of the physical exam at the follow up 2 visit to inform the disease assessment

^4^Serum biochemistry including renal (urea, sodium, potassium, creatinine), liver (AST and ALT, ALP and bilirubin), bone (including serum albumin and calcium), thyroid profiles (including T4, T3, TSH) and lipids, lipase and amylase. Bloods required on the day of treatment can be taken up to one working day in advance

^5^ Full blood count (FBC) to include haemoglobin, white blood cell count, absolute neutrophil, lymphocytes and platelets counts. Bloods required on the day of treatment can be taken up to one working day in advance

^6^Must be serum test at screening. Urine/serum test at week 3 and week 5

^7^ Archival FFPE block to have been collected within 3 months prior to day 1 and should be sent as soon as possible after consent. New tissue biopsy to be collected at week 1 Brachytherapy

^8^Screening scan not required if done within 8 weeks prior to day 1

^9^Baseline imaging assessing for metastases can be done via CT alone or combined PET-CT, and does not need to be repeated if done within 8 weeks prior to day 1

^10^Assessment of fecal urgency (RT-ARD Score) at screening, Radiotherapy week 5 and follow up 2. Assessment of vaginal dilator use at follow up 2 only

^11^To be taken for 7 consecutive days as an outpatient. Tolinapant can be taken with or without food, with the exception of PK days. On days with PK sampling, patients should refrain from eating (including soup) or drinking milk or juice for at least 2 h prior to ingesting study drug(s) and 2 h after ingesting study drug (4 h fasting window)

^12^6 PK samples should be taken across day 4 and day 5 of week 1. Please see Laboratory Manual for further information

^13^Daily cone beam CT imaging will be used to inform the delivery of the radiotherapy, as per standard of care and according to local site policy. External beam radiotherapy should be administered in line with EMBRACE or INTERLACE processes

^14^Brachytherapy schedules may vary according to local site policy in line with Sect. 6.1, however all brachytherapy treatments should be carried out within the 2 week window. Brachytherapy should be image guided (utilizing up to 3 MRI scans and 4 CT scans) as per standard of care and according to local site policy

Prohibited concomitant medication includes other anticancer treatments, including other investigational drugs or therapies, QT prolonging agents (with the exception of palonesetron/Akynzeo when used as part of the chemotherapy antiemetic regime), and strong CYP3A4 inhibitors or inducers.

#### Translational research

Archival fixed paraffin embedded (FFPE) tumour samples will be collected at baseline on all patients. New tissue biopsy will be collected at week 6 (week 1 of brachytherapy). Sections will be cut by histology for the following analyses: H&E stain for pathology reporting, RNA extraction for transcriptomics, Immunohistochemistry (IHC) for IAP and hypoxia status and DNA extraction for investigating the mutational landscape. Cores may also be taken for construction of Tissue Microarrays (TMAs).

Translational blood samples will be collected at every visit, except the final follow-up visit (week 18). One sample will be to study “on-target” effects of tolinapant by measuring cIAP levels in PBMCs. The rest will enable biomarker discovery. This data will enable powering of a future phase II study for biomarker validation, thus enabling both biomarker and clinical development to occur in parallel.

Translational urine samples will be collected at baseline, week 1,3 and 5 (on during RT), week 6 (during brachytherapy) and at both follow up visits. Urinary cell-free DNA (ucfDNA) will be extracted from these samples in order to explore the urine biomarkers associated with treatment response and toxicity.

Collection of translational samples will be part of the main study consent form. Any samples remaining once trial analyses are complete will be stored appropriately for future use, if patient consent allows.

#### Contraception

Women of child-bearing potential must agree to use one of the following methods of contraception effective from the first administration of all study drugs, throughout the trial and for six months afterwards: sexual abstinence, bilateral tubal occlusion, vasectomized partner, a barrier method such as male or female condom (with or without spermicide), cap or diaphragm.

### Data collection and management

#### Plans for assessment and collection of outcomes

Data will be entered into the electronic case report forms (eCRFs) within Medidata Rave by the relevant trained site research team personnel. The data entered will be regularly checked by SCTU for missing or anomalous values and data queries will be automatically or manually generated withing the eCRF.

The Patient Information Sheet (PIS) and Informed Consent Form (ICF) will outline the participant data to be collected and how it will be managed or might be shared, including handling of all Patient Identifiable Data (PID) and sensitive PID adhering to relevant data protection law.

#### Data management

A data management plan has been developed for CRAIN which provides a complete overview of the organisation of data management products and procedures. Participant data will be entered remotely at site and retained in accordance with the current Data Protection Regulations. The Principal Investigator (PI) at each site is responsible for ensuring the accuracy, completeness, and timeliness of the data entered. Only the investigator and personnel authorised by them should enter or change data in the eCRFs.

The participant data is pseudo anonymised by assigning each participant a participant identifier code which is used to identify the participant during the trial. The site retains a participant identification code list, which is only available to site staff.

Data queries will either be automatically generated within the eCRF, or manually raised by the SCTU, if required. All alterations made to the eCRF will be visible via an audit trail.

At the end of the trial, when the last patient has had their last trial visit and after all queries have been resolved and the database frozen, the PI will confirm the data integrity by electronically signing all the eCRFs. The eCRFs will be archived according to SCTU policy and a PDF copy including all clinical and Meta data returned to the Investigator for each participant.

### Oversight and monitoring

The Trial Management Group (TMG) is responsible for overseeing progress of the trial, including both the clinical and practical aspects, and is comprised of clinicians, statisticians and Patient and Public Involvement (PPI) contributors and other SCTU staff. The Chair of the TMG is the Chief Investigator (CI) of the trial. The Trial Steering Committee (TSC) acts as the independent oversight body on behalf of the Sponsor and Funder, and has a majority independent of the trial. The Safety Review Committee (SRC) contains an independent clinician and statistician, alongside the CI and PIs and SCTU staff, and will review and assess the safety and toxicity of all patients at each dose, decide the recommended dose cohort for future patients, decide the maximum number of patients to recruit on that dose, and will determine whether the trial should continue or stop (due to patient safety and/or feasibility issues or the reaching of the MTD). Charters for these groups can be obtained via CRAIN@soton.ac.uk.

The trial may be subject to inspection and audit by University Hospital Southampton NHS Foundation Trust (as Sponsor), SCTU (as the Sponsor’s delegate) and other regulatory bodies to ensure adherence to the principles of Good Clinical Practice (GCP), Research Governance Framework for Health and Social Care, applicable contracts/agreements and national regulations.

A risk assessment for CRAIN has been carried out to identify potential risks and to document any required mitigations that may be required. Following the risk assessment, a trial monitoring plan was developed to detail the expectation for both central and on site monitoring. Data required to inform decision making and to determine the safety of the experimental treatment will undergo full central monitoring. All sites are subject to on-site monitoring, where SCTU will be given direct access to source documentation for verification of data entered onto the eCRF. Monitoring procedures are fully described in the trial’s monitoring plan. Participants’ medical records and other relevant data may also be reviewed during audit of the trial. Details will remain confidential and participants’ names will not be recorded outside the trial site without informed consent.

### Patient and public involvement

There are currently three active PPI members contributing to CRAIN who all have direct experience of cervical cancer, either as a patient or carer. While the trial is active the SRC, TMG and trial steering committee groups will all have PPI representation to assist in the ongoing oversight of the trial. In addition, there has been PPI contribution to the development of the protocol, PIS and ICF. The PPI members have also played a significant role in raising awareness for the trial through various media coverage.

### Statistical analysis

A detailed statistical analysis plan will be developed prior to database lock, and all data and appropriate documentation will be stored for a minimum of 25 years after the completion of the trial. All eligible participants enrolled in the study will be accounted for in the safety analysis. The primary analysis will be based on the principal stratum strategy of the Estimand framework [8] where patients who do not receive any exposure to tolinapant will not be included in the analysis and will be replaced.

DLTs are defined in the *Dose Limiting Toxicology* section.

#### Dose escalation and determination of MTD

The trial will be a single arm, open-label, multi-centre trial that will use the two-stage time-to event continual reassessment method (TiTE-CRM) to find the optimal dose of tolinapant in combination with CRT. This method assigns weights to the amount of time a patient spends in the study without experiencing a DLT and allows for dose decisions to be made without having to wait for full information from each participant enrolled. This allows for a potentially shorter trial duration without sacrificing valuable data. Simulations (see additional file 1) were run to see how this design perfomed under a range of scenarios for the true toxicity levels of the doses and to compare with a standard 3 + 3 cohort design. The optimal dose will be defined as the dose where the proportion of patients experiencing a DLT is closest to the target toxicity rate. DLTs for existing treatment is thought to be in the range 5 to 15%. The target toxicity rate was therefore chosen as 25% as an acceptable rate above this.

Dose levels for tolinapant which will be assessed in the TiTE-CRM are: Level 1: 60 mg; Level 2: 90 mg (starting level); Level 3: 120 mg; Level 4: 150 mg; Level 5: 180 mg. No dose skipping is allowed (i.e., it is only possible to escalate to an adjacent dose). These doses were thought to cover a range of outcomes from a low number of DLTs to those plausibly at, or potentially beyond, the target toxicity rate. The two-stage design allows more control of the dose escalations in the early stages of the study when no DLTs have occurred. The TiTE-CRM model together with emerging safety data will guide escalation decisions but all decisions will be made by the SRC.A maximum of 42 patients will be recruited and treated according to the dose defined by the TiTE-CRM. The first patient recruited will be assigned 90 mg as this will allow for a dose reduction to 60 mg for future patients if the first patients experience excess toxicity. The TiTE-CRM will identify the MTD based on the assessment of dose limiting toxicities (DLTs), this will be used to aid the SRC when determining the recommended phase II dose.

The TiTE-CRM design utilises a one parameter logistic model and linear weights. Meaning that information accrued through the DLT assessment period (12 weeks from the start of treatment) is given weight equal to the proportion of the assessment period that has passed. Any patient experiencing a DLT will be included in the analysis as a full patient equivalent, regardless of the timing of the event. Any patient who discontinues treatment early, for any reason not attributable to a DLT, will be included in the analysis as a patient without a DLT. The Safety Review Committee (SRC) will decide whether an extra patient should be recruited to the current dose based on the amount of treatment they had received.

The treatment schedule is planned to last 7 weeks, with an additional 5 weeks follow up for DLTs. Linear weighting will be used in the TiTE-CRM. For example:

- At trial week 12 (i.e. completion of treatment and 5 weeks of follow-up): in the absence of a DLT this would account for a full patient tolerable outcome.
- At trial week 6: in the absence of a DLT this would account for 0.5 equivalent of a tolerable outcome.

The trial will stop for success if 15 consecutive patients are treated at the current recommendation for the MTD, whilst also ensuring a total of at least 18 patients have been treated at the recommended phase II dose. The trial will stop for safety if there is sufficient evidence to suggest that dose level 1 (60 mg) is too toxic i.e. a posterior probability of DLT of 35% or higher is found reflecting a 10% increase over the expected toxicity rate based on published experience [3]. For more information on the TiTE-CRM design and the stopping rules for the study please see additional file 1.A SRC will be responsible for confirming trial dose escalations within the trial, informed by the dose-toxicity model and safety data. The SRC will decide if patients should be replaced based on the amount of treatment received.

If a fifteenth consecutive participant is treated at a given dose and 18 patients overall have been treated at this dose, no more participants will be recruited. All participants should then be followed up until a DLT or 12 weeks, the model and the MTDare then updated with this information, and this is used to inform the SRC’s decision on the final recommended phase II dose.

At the end of this phase, dose finding data will be summarised descriptively, including baseline characteristics for participants. Dose delivery and toxicities will be reported. Toxicities will be reported by cohort, including type, number, range and worst grade. Complete and partial response rates at 3 months after treatment, measured using Recist v1.1 criteria, will also be summarised descriptively along with relative dose intensity.

The parameters of the dose-toxicity model will be described alongside Bayesian posterior point estimates of the risk of toxicity at each dose and corresponding 95% credible intervals.

All analysis will be done in R, Stata v16 or higher, or SAS v9.4.

### Adverse event reporting and risks

All AEs and SAEs will be reported between the first dose and 30 days after the last dose of tolinapant. Only AEs and SAEs that are considered by the investigator to be related to trial procedures will be reported between the provision of informed consent and the first dose of tolinapant. The SCTU has a UK regulatory compliant real-time SAE reporting process to identify serious AEs and suspected unexpected SAEs that could suspend or stop the trial if warranted.

The most common treatment-related AEs in the phase I aspect of another open-label tolinapant study (ASTX660-01) include nausea, pruritus, vomiting, fatigue and maculo-papular rash. The most common Grade 3 AE related to study treatment was increased lipase.

The most common SARs included pneumonitis, maculo-papular rash and pancreatitis (Astex Pharmaceuticals ASTX660 Investigator Brochure, v7 18th August 2021).

### End of the trial

All patients will be followed up until trial week 18. End of Trial is defined as when the last patient has had their last trial visit and all data to answer the research objectives have been collected. Trial documents will be retained in a secure location during and after the trial has finished. The eCRFs, TMF and other relevant documentation will be archived according to SCTU policy.

## Discussion

If a recommended phase II dose is found and the trial shows tolinapant to be safe and effective in combination with CRT, futher trials may be conducted with the overall aim of providing a synergistic treatment option to improve outcome in a population of relatively young active women. Results of CRAIN will be disseminated to patients and clinical teams through peer-reviewed journal publications and by engaging with relevant patient organisations.

## Trial status

The trial opened to recruitment on 23rd September 2022. A maximum of 42 patients will be recruited over a 24 month recruitment period at at least 6 sites in the UK.

This clinical trial was entered into EudraCT on 30th November 2021 (2021-006555-34) and registered on ISRCTN on 30th August 2022 (ISRCTN18574865). The current protocol is version 5 dated 15th December 2023. REC/MHRA approved protocol amendments are communicated to sites via email and updated trial documentation provided centrally via the trial website. Trial registries will be amended where relevant with explanations for these changes.

### Electronic supplementary material

Below is the link to the electronic supplementary material.

Additional file 1: CRAIN: TiTE CRM Simulations

## Data Availability

Pseudonymised individual participant data within the clinical trial dataset will be available for sharing via controlled access by authorised SCTU staff (as delegated to SCTU by the trial sponsor). Data access can be requested via a SCTU Data Release application form; detailing the specific requirements and the proposed research, statistical analysis, publication plan and evidence of research group qualifications. Please email the completed form to the SCTU Data Release Committee Coordinator at ctu@soton.ac.uk. Data access requests are reviewed against specific eligibility criteria by the SCTU data sharing committee including PPI representation and key members of the trial team. Decisions about requests are made promptly and usually no more than three months after receipt of request. Responses to all data requests, with a clear rationale for any refusals, will be sent promptly to the data requester.

## Abbreviations

AE: Adverse Event
CI: Chief Investigator
CRT: Chemotherapy & Radiotherapy
CTU: Clinical Trials Unit
DLT: Dose Limiting Toxicity
eCRF: Electronic Case Report Form
IAP: Inhibitors of Apoptosis
MHRA: Medicines and Health Care Product Regulatory Agency
MTD: Maximum Tolerated Dose
PI: Principal Investigator
PID: Patient Identifiable Data
PK: Pharmacokinetic
PPI: Public and Patient Involvement
RT: Radiotherapy
SAE: Serious Adverse Event
SAR: Serious Adverse Reaction
SCTU: Southampton Clinical Trials Unit
SRC: Safety Review Committee
TiTE-CRM: Time-To Event Continual Reassessment Method
TMG: Trial Management Group
TSC: Trial Steering Committee
WOCBP: Women of Childbearing Potential
