# Supplementary material for: Dose escalation of tolinapant (ASTX660) in combination with standard radical chemoradiotherapy in cervical cancer : a study protocol for a phase 1b TiTE-CRM clinical trial (CRAIN) in UK secondary care centres
Source: BMC Cancer. 2024 Jun 7;24:702. doi: 10.1186/s12885-024-12310-w (PMC11162013; doi:10.1186/s12885-024-12310-w)
Supplement: Supplementary file 1 — Additional file 1: CRAIN: TiTE CRM Simulations [file 12885_2024_12310_MOESM1_ESM.docx]

**Additional File 1**

**CRAIN: TiTE CRM Simulations**

We have investigated the use of continual reassessment method (CRM) designs for this phase I trial, which aims to determine the recommended dose of ASTX660 in combination with radiotherapy for phase II testing. Given the relatively long assessment period for dose limiting toxicities (DLTs) of 12 weeks, a time-to-event (TiTE) CRM design was deemed appropriate. This design allows for dose escalation without having to wait for full information from every participant enrolled, hence shortening the length of the study. As further discussed later, we have settled on using a two-stage TiTE CRM in order to have more control of dose escalation in the early stages of the study.

This document details the decision-making process and simulation results for our design. The following section (*Design*) lays out the decisions made and initial information to set up the model. We then present the results of simulations (*Performance*) under the defined model. Following this, we compare results with the 3+3 design (*Comparisons*).

All analysis presented was carried out in RStudio or R v3.6.0 unless otherwise stated.

**Design**

We used the one-parameter logistic function. The parameter of this function was assumed to follow a normal distribution. The choice of intercept for the logistic function and the prior distribution for the model slope parameter is discussed below in the *Dose-toxicity model* section.

*Number of doses*

Five doses were chosen, from 60mg to 180mg in fixed dose 30mg increases, according to previous results from Astex, where 180mg was recommended as the phase 2 dose when used as monotherapy (ASTX660 Investigator Brochure). These doses were thought to cover a range of outcomes from low a number of dose-limiting toxicities (DLTs) to those plausibly at, but potentially beyond, the target toxicity level (TTL).

*Targeted toxicity level*

Given that current radiotherapy treatment is given with curative intent, it was deemed important not to create an excessive number of toxicities that could disrupt current treatment. DLTs for existing treatment is thought to be in the range 5 to 15% (Naik et al., 2016). The TTL was therefore chosen as 25% as an acceptable rate above this.

*Prior probabilities*

Limited data on the toxicity at different doses of the drug was available from a single phase two study. This study was undertaken on a small number of patients given ASTX660 alone (ASTX660 Investigator Brochure). Given the uncertainty of the DLT rates at each dose, the choice of prior probabilities for each level was based on the methods reported by Lee & Cheung (2010). This approach offers a way of simultaneously determining the prior probabilities and the variance of the prior distribution of the logistic model slope parameter (see *Dose-toxicity model* section). The method requires specification of the TTL, number of doses, the expected maximum tolerated dose (MTD), and a value for the indifference interval, δ. This interval represents what DLT rates are acceptable when selecting a dose for a new participant.

We assessed a range of δ (0.01 to 0.15 in intervals of 0.01, based on Lee & Cheung) and chose δ=0.04 as the prior probabilities most closely resembled the limited prior information; primarily, the lowest dose for this δ had a DLT rate in the range of 5-15%, matching what would be the expected rate even without any ASTX660. This was carried out using the *getprior* function in the *dfcrm* package in R (*v2.1*; Cheung, 2019). The final prior probabilities chosen are given in Table 1. This value of δ gave similar initial probabilities for each dose being chosen as the MTD (18-22%).

| **Dose (mg)** | **Prior probability of DLT (%)** |
| --- | --- |
| 60 | 11 |
| 90 | 18 |
| 120 | 25 |
| 150 | 33 |
| 180 | 42 |

***Table 1****: prior probabilities of DLTs for each dose*

*Dose-toxicity model*

To finalise the initial model, we required an intercept for the logistic function and parameters for the prior distribution of the slope parameter. We modelled the slope with a normal distribution, using the R default prior mean of 0. We again used the Lee & Cheung approach to determine a least informative prior variance; this ensured that the prior probability of selecting a given dose as the MTD was approximately equal across all doses. The prior variance that achieved this was 0.186.

We next assessed the choice of intercept, based on the width of credible intervals for the prior slope and how well they represented clinical uncertainty. We plotted the credible intervals across a range of values between -5 and +5 and found the default value of +3 to be acceptable; this was based on what were thought to be clinically plausible levels of uncertainty (e.g., approximately 20 to 60% DLT rate for the highest dose) and having similar levels of uncertainty across all doses. The plot using this intercept is given in Figure 1.

***Figure 1:*** *plot of logistic function based on prior distribution of model parameter. The solid line represents the mean of the distribution; the dashed lines represent the 90% credible interval. Vertical dotted lines are equivalent to the doses being evaluated; the horizontal dotted line is the TTL.*


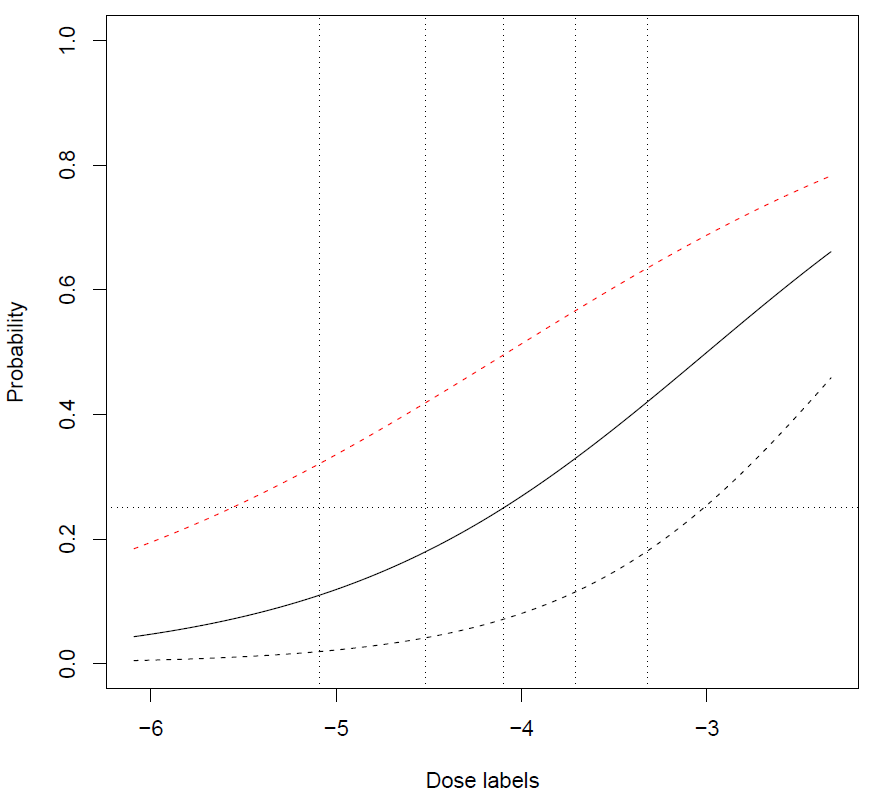


*Safety modifications and stopping rules*

The second dose (90mg) was chosen as the starting dose. This dose was thought to have DLT rate below the TTL, while also allowing for de-escalation, if needed. No dose skipping was allowed (i.e., it was only possible to escalate to an adjacent dose).

Two stopping rules were implemented. Firstly, once a fifteenth consecutive participant was recommended a given dose, no more participants were recruited. In the simulation results, this dose was then chosen as the maximum tolerated dose (MTD); however, we would anticipate following all participants up until a DLT or 12 weeks, updating the model with this information, and using this to inform the SRC’s decision on the MTD. Fifteen was chosen as the limit as, under simulations for a number of scenarios, this meant fewer than 10% of trials would have chosen an MTD different to the dose with fifteen consecutive participants. We initially chose eight (as per the original grant application), but more than 40% trials would later choose a different MTD if allowed to continue recruitment, which markedly reduced the operating characteristics (in terms of choosing the correct MTD). The second stopping rule was to stop the trial if the DLT rate was estimated to be 35% or more for the lowest dose. This had very little impact on the operating characteristics other than for scenarios where toxicity was very high, with the stopping rule leading to a proportion of studies previously choosing the lowest dose now recommending no dose. The figure of 35% was chosen as it is 10% above the upper limit of what was deemed an acceptable toxicity rate, allowing for some overestimation of the DLT rate for the lowest rate if it was still within the acceptable range of DLT rates.

*Other considerations*

TiTE CRM requires specification of the weighting of the likelihood, i.e., how much a participant contributes to the recommended next dose when the DLT assessment period is not complete. We used a linear weighting, meaning that information accrued through the DLT assessment period is given weight equal to the proportion of the assessment period that has passed.

The original funding application specified that two tolerable outcome equivalents would be required before escalating; however, this was found to significantly extend the study length and to reduce the efficiency of the study. As further discussed below, this was replaced by a two-stage design, where we maintain control of escalation early in the study but do not enforce any restrictions on tolerable outcome equivalents. This approach allowed for more flexible dose recommendations, while maintaining safer escalation at a time when the prior information may dominate the incoming data.

**Performance**

*Set-up*

Model performance was assessed via simulations carried out in R using the *titesim* function in the *dfcrm* package. A total of 1,000 simulations were used throughout, except for the final results which used 2,000 simulations for additional precision. Performance was assessed using how frequently the MTD was chosen, the number of participants, the number of DLTs experienced, and the length of the study.

Multiple scenarios were considered: these ranged from very low toxicity across the doses (14% DLT rate at highest dose) to very high toxicity (30% at lowest dose). Each dose was the MTD in at least one scenario (scenarios 1 to 5 in Table 2). Performance where the true DLT rates matched the prior distribution was also assessed (scenario 6), along with two further scenarios (7 and 8) representing the upper and lower prior DLT rates anticipated.

|  | **Scenario** | **MTD (dose)** | **DLTs (%) by dose (mg)** | | | | |
| --- | --- | --- | --- | --- | --- | --- | --- |
|  |  |  | **60** | **90** | **120** | **150** | **180** |
| 1 | High toxicity | 1 | 30 | 50 | 70 | 80 | 90 |
| 2 |  | 2 | 15 | 25 | 35 | 45 | 55 |
| 3 |  | 3 | 5 | 10 | 25 | 40 | 45 |
| 4 |  | 4 | 14 | 15 | 17 | 25 | 45 |
| 5 | Low toxicity | 5 | 10 | 11 | 12 | 13 | 14 |
| 6 | Prior | 3 | 11 | 18 | 25 | 33 | 42 |
| 7 | Lower | 1 | 10 | 12 | 15 | 18 | 25 |
| 8 | Upper | 2 | 17 | 27 | 38 | 48 | 58 |

***Table 2****: Scenarios tested in simulations.*

*Iterations*

Under these scenarios, we evaluated the performance of our TiTE CRM design. Having established the model’s performance in terms of percentage of correct identification of MTD etc. we next investigated individual trial behaviour by looking in detail at the simulations.

We noticed that participant 2 was always escalated to dose 3 no matter the outcome for participant 1, suggesting that our prior had too much influence. To counter this, we implemented a two-stage TiTE CRM in order to maintain closer control of the escalation early on when little data was available.

The first attempt was to fix the first two participants to receive dose 2 and to be observed for the full assessment period (12 weeks). While this prevented the second participant being recommended dose 3, the simulation results showed that if participant 3 was on dose 3 and no DLTs had been observed then the dose would be immediately escalated for participant 4 even with only minimal information from participant 3.

To prevent this rapid escalation, we tweaked the design so that the first two participants receive dose 2, and, if no DLTs in the first 6 weeks, the next two participants receive dose 3 (and then the same for dose 4). This has much better properties for reducing potentially unsafe early escalation, with only a minor impact on the operating characteristics (in terms of choosing the correct MTD; the two-stage design appeared to reduce the average number of DLTs across all scenarios).

This approach was felt to be an acceptable compromise that ensured we didn’t escalate towards higher doses without undue risk, while also not significantly lengthening the trial duration.

*Final two-stage TiTE CRM Design*

The initial stages of our final design using a two-stage TiTE CRM are given in Figure 2 below. The first two participants will be given dose 2 and followed up for 6 weeks; if no DLT occurs, the next two participants will receive dose 3, and so on. Once a DLT occurs, the CRM model will be used to recommend the next dose.

The recommended next dose following a DLT is not fixed; for example, following escalation to dose 3 after the first two participants, one DLT may result in dose 2 or dose 3 being recommended for participant 5. The recommended dose will depend on the dose at which the DLT occurred.

***Figure 2:*** *dose transition pathways for the two-stage TiTE CRM.*


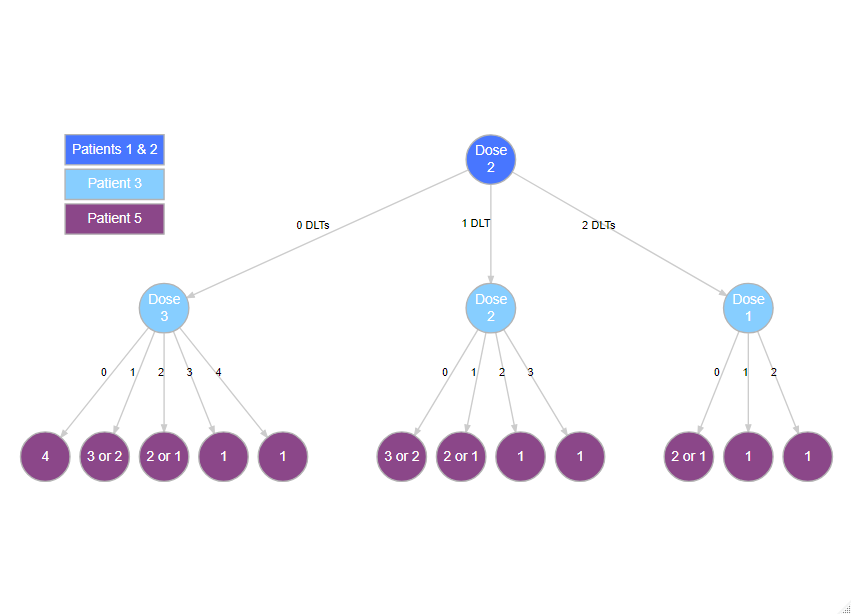


*Results*

Table 3 shows the percentage of times the correct MTD was chosen, as well as how often a dose within the 15 to 35 % range was chosen.

***Table 3:*** *Green cells represent the true MTD for that scenario.* We present the number of simulated trials that stopped due to the enforced stopping rules (15 consecutive participants on the same dose, and an estimated DLT rate of >35% for the lowest dose), as well as the average number of participants, the average given each dose, and average number of DLTs. Results for chosen MTDs are presented in Table 3.

| **Scenario** | **Dose (mg) chosen as MTD (%)** | | | | | | **% times chosen MTD within 15-35%** |
| --- | --- | --- | --- | --- | --- | --- | --- |
|  | **None*** | **60** | **90** | **120** | **150** | **180** |  |
| 1 | 22 | 70 | 8.3 | 0.1 | 0.0 | 0.0 | 92 |
| 2 | 0.4 | 16 | 52 | 28 | 3.9 | 0.2 | 96 |
| 3 | 0.0 | 0.2 | 18 | 62 | 19 | 1.7 | 62 |
| 4 | 0.1 | 1.3 | 10 | 31 | 49 | 8.7 | 80 |
| 5 | 0.1 | 0.2 | 3.3 | 8.1 | 23 | 66 | - |
| 6 | 0.1 | 3.3 | 26 | 45 | 22 | 4.1 | 93 |
| 7 | 0.1 | 0.4 | 5.3 | 17 | 37 | 41 | 94 |
| 8 | 0.6 | 23 | 53 | 21 | 2.2 | 0.1 | 76 |

***Table 3:*** *Percentage of times each dose was selected as MTD by two-stage TiTE CRM and percentage of times chosen MTD was within the range 15-35% DLT rate. Green cells represent the true MTD. *None means trial was stopped as no dose was deemed safe (i.e., lowest dose with DLT rate >35%)*

Between 22 and 34% of the simulated trials stopped early due to 15 consecutive participants being recommended the same dose, except for scenarios 1 (high toxicity) and 5 (low toxicity, where 47 and 46%, respectively, were stopped for this reason. All scenarios averaged 27-29 participants, except scenario 1 (mean 24 participants), with mean trial durations of 18.4 to 19.7 months (and 15.8 for scenario 1). Mean DLTs over all scenarios was 6.9, with up to 10 for more toxic scenarios and 3.5 for less toxic scenarios.

We note that the percentage of correctly chosen MTD is not as high as we would like in some scenarios. We believe this may be due to a number of factors, most prominently the DLT rates at neighbouring doses being too similar for the model to distinguish between them. The comparison of scenarios 3 and 6 highlight this point, where true DLT rates of adjacent doses are further apart in scenario 3, resulting in a noticeably increased rate of determining the true MTD. For scenarios where the rates are close together, it may be that the sample size is too small to determine the MTD with any more accuracy than we currently have.

Changing the prior variance for the logistic model parameter changed the frequency with which the model determined the correct MTD; for example, choosing a value that led to higher prior probabilities of a particular dose being the MTD meant that dose was more often chosen as the MTD by the model. However, what improves performance in one scenario led to decline in performance in other scenarios. Given the lack of prior information, we stayed with our choice of uninformative prior.

**Comparisons**

Although some of the simulations show the model does not perform as well as we would like, we are still confident that this is the best design for the study. We compared the results of our two-stage TiTE CRM simulations with those of a 3+3 design. Using a SAS macro developed by Pillai et al. (2015) for simulating 3+3 trials, we once again ran our eight scenarios and tabulated the number of times the correct MTD was chosen. To allow direct comparison, we again started the trial on dose level 2 and allowed the trial to be stopped if the lowest dose was too toxic.

| **Scenario** | **Dose (mg) chosen as MTD (%)** | | | | | | | **% times chosen MTD within 15-35%** | |
| --- | --- | --- | --- | --- | --- | --- | --- | --- | --- |
|  | **None*** | **60** | **90** | **120** | **150** | **180** |  | |  |
| 1 | 51 | 39 | 11 | 0.1 | 0.0 | 0.0 | 90 | |  |
| 2 | 10 | 36 | 34 | 16 | 3.1 | 0.9 | 80 | |  |
| 3 | 0.5 | 10 | 39 | 35 | 11 | 4.5 | 35 | |  |
| 4 | 2.9 | 17 | 19 | 27 | 26 | 8.2 | 53 | |  |
| 5 | 1.0 | 9.4 | 11 | 10 | 13 | 55 | - | |  |
| 6 | 3.6 | 22 | 34 | 20 | 13 | 5.0 | 67 | |  |
| 7 | 2.0 | 12 | 15 | 18 | 22 | 32 | 72 | |  |
| 8 | 11 | 38 | 36 | 13 | 2.0 | 0.3 | 74 | |  |

***Table 3:*** *Green cells represent the true MTD for that scenario.* ********None means trial was stopped as no dose was deemed safe (i.e. too many DLTs observed at the lowest dose)*

For direct comparison, Figures 3-5 shows the percentage of times a dose was chosen as the MTD for the two-stage TiTE CRM and the 3+3 design under scenarios 6-8. In these examples, the 3+3 design frequently chooses a dose that is too low as the MTD. A 20% difference in choice of correct MTD is largely typical of other scenarios. This supports the choice of the two-stage TiTE CRM as an appropriate design for this study.

***Figure 3:*** *percentage of times each dose was chosen as MTD under each model for scenario 6, where the true MTD is dose 3 (120mg).*

***Figure 4:*** *percentage of times each dose was chosen as MTD under each model for scenario 6, where the true MTD is dose 5 (180mg).*

***Figure 5:*** *percentage of times each dose was chosen as MTD under each model for scenario 6, where the true MTD is dose 2 (90mg).*

**References**

Lee & Cheung (2010). Calibration of prior variance in the Bayesian continual reassessment method. *Statistics in Medicine.* DOI: 10.1002/sim.4139

Naik et al. (2016). Comparison of dosimetric parameters and acute toxicity of intensity-modulated and three-dimensional radiotherapy in patients with cervix carcinoma: A randomized prospective study. *Cancer Radiotherapie*; v20(5), pp 370-6.

Pillai et al. (2015). Establish the Maximum Tolerated Dose in Phase-I Trials using 3+3 Method. <https://www.phusewiki.org/docs/Conference%202015%20SP%20Papers/SP04.pdf> (last accessed 28/02/2020).

Wheeler et al. (2019). How to design a dose-finding study using the continual reassessment method. *BMC Medical Research Methodology*; v19.
